# Perspectives of community processes in establishing community-based mental health services in Hong Kong: a case study

**DOI:** 10.1186/s13033-022-00518-x

**Published:** 2022-01-29

**Authors:** Vincent W. P. Lee, Daniel W. L. Lai, Xiaoting Ou

**Affiliations:** 1 Department of Applied Social Sciences, The Hong Kong Polytechnic University, Room GH410, Kowloon, Hong Kong; 2 Faculty of Social Sciences, Hong Kong Baptist University, Kowloon, Hong Kong; 3 Department of Sport, Physical Education and Health, Hong Kong Baptist University, Kowloon, Hong Kong

**Keywords:** Hong Kong, NIMBY, Mental health stigma, Mental health community service, Public consultation, Community education

## Abstract

**Background:**

Integrated Community Centres for Mental Wellness (ICCMWs) provide district-based community support services for patients discharged from mental health facilities and other residents in Hong Kong. However, selecting locations for these community centres is challenging primarily because of community opposition, which has introduced barriers to responses to service users’ interests and the operations of individual centres. This study examines public preferences for conflict resolution options, evaluates the feasibility of different consultation approaches, identifies effective methods for reducing public opposition and recommends possible approaches to public consultation and location selection.

**Methods:**

A total of 74 individual interviews were conducted with key informants, including government personnel, service operators, politicians, resident representatives, community activists and service users. These interviewees were asked about their knowledge, experiences and perspectives of centre location selection processes.

**Results:**

Interviews revealed that neighbourhood consultations for establishing community centres for mental wellness are time-consuming and did not yield a consensus of support from residents. In some instances, the government may decide to delay or withdraw location selection plans because of strong public opposition mainly because of bias and misunderstanding towards service users. However, the role of local politicians in mediating neighbourhood opinions and the government’s determination in planning location selection are essential for successfully selecting ICCMW locations.

**Conclusion:**

Government departments should develop stronger collaboration to study target neighbourhoods and lobby stakeholders at earlier stages. Such investigations should involve identifying key stakeholders, the political and social dynamics of controversies and community demographics. A protocol specifying a time frame should be implemented to facilitate smooth and effective public consultation and community mental health service location selection processes.

## Background

Studies consistently revealed unwelcome and discriminatory attitudes against people with mental illness [1, 2]. In addition, actions have been taken to stop those with mental illness from receiving the services and support they require. The establishment of facilities and services for people with mental illness has been objected to and challenged by residents in Western societies [1–5]. Opposition towards the establishment of facilities and services for people with mental illness continues, even in Hong Kong, despite the city’s progressive mindset and outlook [6]. This study examined community perspectives towards the establishment of community-based mental health facilities in Hong Kong. That is, the study examined public preferences for conflict resolution options and evaluated the feasibility of different public consultation approaches. Moreover, this study identifies effective methods for reducing public opposition and recommends possible approaches to addressing the community processes required to establish such facilities or services.

### Discrimination challenges: the ‘Not in My Backyard’ phenomenon

The ‘Not in My Backyard’ (NIMBY) phenomenon is the key concept underpinning opposition to establishing facilities or services for people with mental illness. NIMBY is a common challenge in development processes and is associated with the construction of public service facilities not favoured by residents of particular neighbourhoods, known as ‘locally unwanted land uses’ [5, 7–9]. When such projects are proposed, residents may take sceptical or even hostile approaches to their construction. The arguments presented in opposition involve issues related to child safety, local traffic capacity, lack of complementary professional support services and more urgent needs for services for other social groups, such as children or older people [7]. However, the fundamental preference of these oppositions is that the service or facility would be more suitable for other neighbourhoods considered to have more pressing needs [9]. The same vicious opposition cycle may repeat when the intention of establishing the facility in another neighbourhood is revealed. NIMBY-based opposition to proposed public service facilities may result in reduced access to critical services or excessive travel requirements for service users, including a degradation of community relations that might negatively affect service users [7].

NIMBY mentalities may be related to a lack of public knowledge and understanding of mental illness and the approaches to public consultation and engagement. For example, Takahashi found that in the United States, most respondents reported that they were not very well informed about mental disability and rarely had personal experience with mental health care. Moreover, the information they obtained often originated from popular media [10]. Therefore, service planners and providers should address the mechanisms influencing the formation of public frames of reference concerning risks and explore how new perceptions can be constructed and perpetuated to address controversies involved with the establishment of mental health facilities [1, 4, 7]. However, with years’ worth of enhancements in the public’s understanding of the myths about mental illness and the needs of people with mental illness, communicating with local residents through public meetings or consultations cannot entirely change biases or oppositional perspectives [1, 9, 10]. How public consultations are conducted can encourage or discourage the NIMBY mentality in various communities. Cowan examined opponents’ perspectives in response to the relocation of mental health services to communities in Scotland. The author found that the obstacles and strong oppositional sentiments could be related to public consultation processes [11]. For example, in Scotland, consultation was intended to offer citizens an opportunity to express their views and alleviate any anxieties. However, the official guidelines did not indicate the content or form of the perspectives likely to be encountered. Therefore, nothing was mentioned regarding the characteristics of the consultation process in the context of the arguments that were likely to be deployed in debates about the proposed establishment of a facility. The authorities did not indicate how practitioners should enter and engage in these debates or respond to opposition. Although consultation is often relatively unproblematically defined in terms of its function, the specific nature, process and outcome of consultation can be more challenging and complex. Other studies identified oppositions to mental health facilities or services in community settings [4, 5].

### International experiences in establishing controversial social facilities

The literature on approaches to address the establishment of community mental health facilities in other world jurisdictions was searched. Some more developed countries, such as Australia, New Zealand and Canada, have resorted to using human rights legislation to form the basis for establishing mental health and other controversial community facilities [12–16]. These legislations explicitly protect the statutory rights of people with disabilities and mental illness to receive residential and other social services.

Some countries, such as Singapore, have clear legal protocols for land zoning specifically for establishing social services, emphasising social integration rather than establishing separate premises for mental health services [17]. Strong legal binding strategies are used to achieve the goal of establishing community-based services without requiring the use of local public consultation.

In some nearby jurisdictions, such as Macao and Taiwan, negotiation and collaboration with community stakeholders are adopted when establishing mental health facilities and other sensitive community services. No official standardised guidelines or protocols exist for public consultation as most of these facilities are housed in private properties. Service providers and patients’ groups generally have to negotiate with owners’ and local residents or community organisations in the event of opposition [18–20].

Some countries, such as Japan and Korea, have less developed policies on the rights of people with mental illness because of taboos and stereotypes. As the use of in-patient treatment is the mainstream approach, no official policies and protocols exist for establishing community mental health facilities [21–24].

### Integrated community centres for mental wellness in Hong Kong

In the 2009–2010 policy address of the Hong Kong government, the increasing needs and demands for community-based mental health services were recognised, resulting in the decision to establish territory-wide Integrated Community Centres for Mental Wellness (ICCMWs). This policy emerged from recognising the benefits of a medical–social collaborative model echoing the global trend of providing community-level care to promote recovery, following several high-profile fatal incidents involving people with mental illness [25, 26].

The objective of the ICCMWs is to provide district-based, accessible community support and social rehabilitation services, including early prevention and risk management for former patients of mental health facilities, people with suspected mental health problems, families and carers of patients with mental health problems and social rehabilitation services for other residents living in the neighbourhood through a single-entry point [27–30]. These ICCMWs provide community-level prevention, detection and recovery services for mental illness, in contrast to treatment for patients with severe mental health problems, which is the responsibility of public hospital-based psychiatric services. Community education, support groups, case management, counselling, drop-in services, outreach services and recreational and volunteer support activities are examples of the services provided through ICCMW by professionals, such as social workers, clinical psychologists and occupational therapists. Operators of the ICCMWs are required to achieve the performance standards in accordance with the team size in the specified districts as listed in the Funding and Service Agreement by the Social Welfare Department (SWD) [31, 32].

Service providers in several districts have established permanent sites, mainly because of strong public opposition. Others are still renting or borrowing temporary service spaces that do not meet the service provision specifications, thereby hindering service delivery and causing inconveniences for users. As of August 2021, only 17 of 24 ICCMWs have been established at permanent venues, and seven of which are located at temporary service delivery sites (four have been designated permanent premises, and three are waiting for a suitable site, with no expected completion date). This case may further hinder the establishment of additional community-based mental health facilities [33] despite the substantial demand for the services. In 2018–2019, the number of ICCMW users (as of December 31, 2018) had reached 25,836, alongside 4011 family caregivers [31].

### Establishment processes

The SWD of Hong Kong is responsible for identifying sites appropriate for permanent ICCMWs in all 18 districts. Although the SWD is responsible for the planning and implementation of ICCMWs, differences have been observed in attempts to secure permanent premises amongst the various districts and communities. The SWD and relevant service providers have encountered numerous challenges and obstacles during these processes. *Hong Kong Planning Standards and Guidelines* (HKPSG) [34] stipulates that an ICCMW should be located where the population is concentrated and where public transport is easily accessible. For the convenience of their service users, centres should be barrier-free and located near other social welfare facilities.

HKPSG also provides precise specifications for conducting public consultations before a new community service facility is established. These comprehensive provisions cover issues, such as formulating consultation plans in the early stages of planning, identifying the people and groups to be consulted, the information provided to stakeholders and responses to consultees’ concerns.

However, several obstacles to securing permanent ICCMW premises in numerous districts have emerged mainly because of public opposition, primarily caused by discrimination and stigmatisation. Relevant studies provided evidence on stigmatisation and discrimination against people with mental illness and former patients in Hong Kong [4, 5, 11].

## Methods

Evidence supporting the prevalence of stereotypes and discrimination is currently lacking, specifically in terms of how such beliefs and perspectives influence community response processes. In addition, it is a critical process for government officials to consider the impacts of the inputs from neighbourhood residents when finalising decisions related to the establishment of service centres. Thus, this study examined the perspectives of various key informants regarding the establishment of ICCMWs. The specific research objectives were as follows:

- To understand the rationales for supporting or opposing the establishment of facilities
- To examine public preferences for conflict resolution options

The analytical framework of this study is based on the framework for assessing the impact of the NIMBY phenomenon on community health developed by Sénécal and Reyburn [3]. The framework aims to improve the understanding of the capacity of a community to consider tensions and address issues related to planning practices and environmental management. The major components of this framework are the following: (1) the NIMBY phenomenon, including different types of requests by various stakeholders; (2) NIMBY and community health concerns and (3) the results of public engagements and negotiations. This framework uses the tensions expressed by the community to improve the quality of life and the environment and determine methods for managing disagreements amongst stakeholders. Based on the social and environmental backgrounds of issues related to the selection of ICCMW locations in Hong Kong, Sénécal and Reyburn’s framework was modified to focus on three primary dimensions. Firstly, the rationales of residents and community stakeholders for supporting or opposing the locations selected for ICCMWs in various neighbourhoods were studied. Secondly, the strategies preferred by the public to resolve conflicts and controversies regarding the locations selected for ICCMWs were identified. Thirdly, the most effective approaches for reducing public opposition regarding the selection of ICCMW locations were determined.

A thematic analytical approach was employed to identify the themes and perspectives of the aforementioned research objectives in the context of the perspectives of the research participants.

### Data collection and analysis

Interviews with key informants were conducted between September 2017 and May 2018. In this study, the interviews focused mainly on the interviewees’ experience with existing consultation mechanisms and their perspectives regarding the establishment of ICCMWs, including their recommendations for improving consultation mechanisms and approaches for reducing public opposition to the establishment of ICCMWs. Additionally, for ICCMWs that had not secured permanent premises, the interviewees were asked about the engagement process, different stakeholders’ roles, circumstantial uniqueness and their previous service operation experiences, current challenges and plans for service development and selecting locations for permanent premises. With the consent of the interviewees, the interviews were audio-recorded and transcribed verbatim. Thematic analysis was employed to identify the codes and messages that constitute the critical themes identified through the following steps [35]: (1) search for themes, (2) review themes, (3) define and name themes and (4) prepare documentation [35].

### Targets and sampling

To identify multiple and diverse perspectives, purposive sampling was employed to recruit research participants from different social sectors related to the establishment of ICCMWs, including government personnel, politicians, service providers (agency leaders or service managers of ICCMWs), community members (local residents or community leaders) and ICCMW service users. A purposive sampling method was used to identify participants. This nonprobability sampling method involved selecting potential participants based on the researcher’s judgement regarding usefulness and representativeness [36]. Snowball sampling methods were adopted for recruiting key participants. Specifically, potential key participants were identified through publicly available channels and personal networks. For example, connections were made through the CEOs or service directors of 11 ICCMWs through a provider with whom the research team had a long working relationship. Specific colleagues responsible for ICCMWs from other agencies were also introduced to the research team.

A total of 74 interviews were completed (Table 1). Interviews were conducted semi-structured, which enabled the interviewees to share their knowledge and perspectives with minimal limitations to maximise the amount of detailed information obtained. Each interview lasted for approximately 45 min. Table 1 presents the key participant categories, the number of in-depth interviews conducted during data collection and the number of people or cases covered during the interviews.

**Table 1:** Number of informants from different stakeholder groups (September 2017–May 2018)

Key respondents	Number of interviewees
Government personnel	13 (including representatives from the SWD and the Housing Department)
Managers of ICCMWs	14
District Council (DC) members and Legislative Council (LegCo) members	19 (15 DC members and 4 LegCo members)
Community leaders and residents (Mutual Aid Committees or residential organisation representatives; non-member volunteers from the community)	20
Service users	8
Total number of key participant interviews	74

## Results

The analysis of the key interviews revealed themes associated with the four research questions. In addition, an in-depth analysis of four ‘typical’ successful cases and two unsuccessful cases regarding the selection of ICCMW locations was conducted to improve the understanding of the specific factors affecting the ICCMW establishment process and the effectiveness of different consultation approaches.

### Rationales for support and opposition

The interviews revealed various rationales associated with support for and opposition to the establishment of ICCMWs based on fear or misunderstanding regarding mental illness and mental health services, acceptance of differences, experience with mental illness and a desire to support mental health patients.

Firstly, community support or opposition is based on perceptions of danger associated with mental illness, the patients of mental health facilities and their potential implications for the neighbourhood. These perceptions largely depend on public attitudes towards mental health patients and (mis)understandings of mental health, recovery and the services they receive. Key participants indicated that acceptance of patients and former patients of mental health facilities has significantly improved in Hong Kong because of the efforts of service providers, service users and volunteers to engage with the public. However, most community members believed that discrimination still exists. Key interviews and news reports suggested that residents oppose location selection plans largely because they did not understand the nature of the services provided by ICCMWs or were not well informed about ICCMW users and the scope of services provided in their neighbourhoods. Mass media often report negative news regarding mental illness and isolated incidents involving patients of mental health facilities, creating an impression that they could be dangerous, which triggers negative sentiments amongst neighbourhood residents. Certain DC members still express serious concern about the government acting against residents’ oppositions. Several such DC members claimed that their obligations and duties are to represent the opinions of their local constituents.

Secondly, levels of support or opposition were based on acceptance of differences, including mental illness. One key participant said that ‘in Hong Kong, everyone needs a home, so some people with special needs require mental health rehabilitation facilities in the community’. These perspectives were influenced in part by residents’ sociodemographic backgrounds. Some DC and neighbourhood association members indicated that districts with residents from diverse sociodemographic backgrounds and cultures might be more likely to accept different types of people, including those with mental illness. They may be more likely to support the establishment of an ICCMW.

All DC members and legislators that were interviewed agreed with the general principle and benefits of ICCMWs and the establishment of ICCMWs in their constituencies if required. Some, who were also social workers, shared personal experiences of helping patients and former patients of mental health facilities and their families. They highlighted the unavailability of mental health rehabilitation and community education services in Hong Kong. They emphasised the need for ICCMWs in the community, explaining that permanent sites could achieve the greatest outcomes for service users, thus improving convenience, sense of belonging and privacy. Almost all of them agreed that the government’s determination to successfully establish ICCMWs despite public opposition during the consultation process was essential. Although many may oppose plans for establishing ICCMWs, prolonging the consultation process is not appropriate. After selecting a site, the government should proceed with the location selection process and avoid prolonged delays or withdrawal from the decision. A more structured protocol should be formulated to guide the establishment of ICCMWs in a more orderly, consistent and transparent manner. Government departments and service providers should not be limited to engaging in activities and interactions with community leaders. Additional opportunities should be provided for residents to raise concerns and government officials and service providers to address and respond to such concerns.

### Public preferences for conflict resolution

When responding to opposition to plans for establishing ICCMWs, the approaches to addressing and resolving disagreements, tensions and conflicts must be considered. Key participants expressed different perspectives and preferences regarding conflict resolution options.

Firstly, residents emphasised the importance of decision-making transparency in selecting ICCMW locations and in response to consultation processes. For example, according to the key informants and the analyses of case studies, how the SWD responds when they encounter opposition is currently not satisfactory. Numerous participants from the social welfare sector complained about the lack of transparency in the SWD district office regarding location selection. One participant indicated that the SWD had posted a list of available premises on the website in the past but not anymore. Nongovernmental organisations (NGOs) must now independently contact the Housing Department offices to search for empty premises. Additionally, in some cases, location selection plans were either postponed for several years or simply withdrawn, which affected service development and the interests of service users. Residents were dissatisfied with the lack of transparency during consultations, possibly resulting in negative comments and stigmatisation.

Secondly, opposition and conflict can be addressed through support and engagement by neighbourhood associations, local DC members and other organisations. For example, an ICCMW was smoothly established in a New Territories neighbourhood because of strong support from residents’ representatives. DC members, service providers and the SWD conducted active community outreach activities to promote understanding and acceptance. Another ICCMW was established with little opposition after local government officials and local politicians (i.e. the District Social Welfare Officer and District Councillor) explained to local residents regarding service user needs and the community’s obligation in the location selection process. Similarly, the stance of individual local politicians was critical in the establishment of ICCMWs, with some participants indicating that if the District Councillors proactively endorse the plan, residents would generally be willing to accept.

Similarly, residents’ representatives play a key role in successful consultation processes by disseminating unbiased information to residents and explaining both location selection plans and the nature of the services provided by the ICCMW. However, conflict resolution processes may be affected by the conflicting political stances of local leaders. For example, in one district, some DC members had contrasting opinions and proposed opening the centre elsewhere (potentially because of the DC elections and concerns about how support for the proposed ICCMW location might affect election results).

Finally, effective conflict resolution and the successful establishment of ICCMWs involves efficient responses to public concerns and the progress of location selected by the government. Residents’ concerns should be addressed in a responsive, patient and timely manner to ensure that they feel respected and mitigate negative impressions towards establishing an ICCMW in their neighbourhood. Government departments, for example, should proactively follow up with the opposition and address residents’ concerns. In addition, the consultation process itself should include opportunities for residents to raise concerns and for government officials and service providers to address and respond to such concerns.

### Effectiveness of different consultation approaches

The examination of successful and unsuccessful ICCMW location selection cases illustrated specific, evidence-based elements of effective and ineffective approaches to public consultation.

**Fig. 1:**
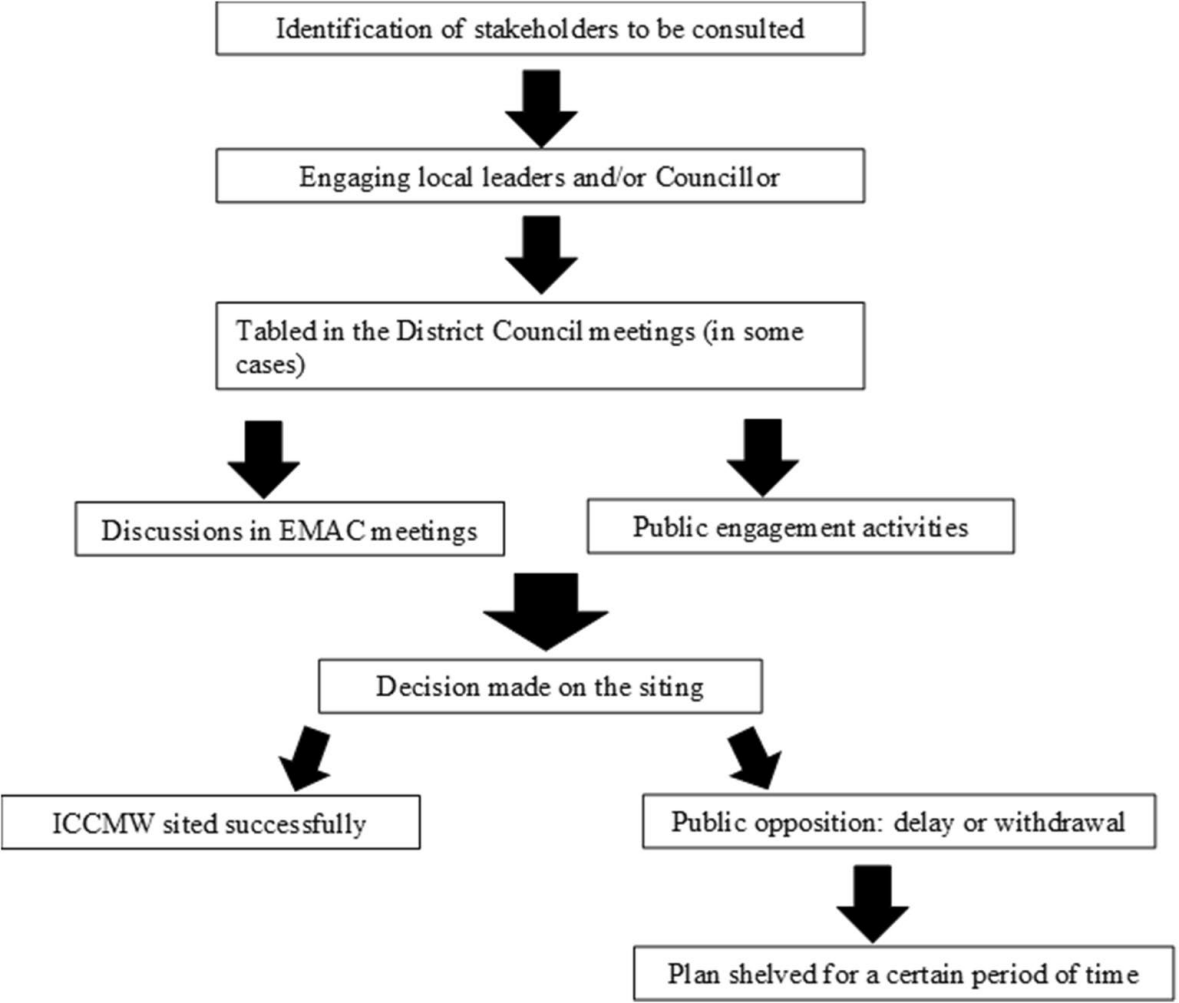
Location selection process for ICCMWs. This figure illustrates the current location selection process for ICCMWs, which starts with the identification of stakeholders from the neighbourhood in which the ICCMW is to be established. These stakeholders may include DC members, residents’ representatives, and religious organisations. The next step involves engaging these local leaders and the DC representing the surrounding neighbourhoods to more thoroughly understand the community dynamics and the initial attitudes of the residents towards the proposed ICCMW. If no strong opposition is expressed by the community stakeholders, the project may be presented for discussion in the DC general meetings. However, this is not applicable to all cases, and it depends on the number of DC members that are interested in discussing the issue. Subsequently, the SWD and the service provider should launch a series of consultation activities in the neighbourhood, including meeting with the members of the Estate Management Advisory Committee and engaging with the residents of the neighbourhood by through leisure activities, seminars on mental wellness, and other kinds of community development exercises. After collecting the opinions from the community leaders, politicians, and individual residents, the SWD decides whether the proposed ICCMW could be successfully established in the neighbourhood. The degree of public opposition is a crucial factor when making such a decision. The location selection is delayed or withdrawn if public opposition is substantial

Firstly, regarding consultation periods, shorter consultations are more effective, and unsuccessful cases were more likely to involve a prolonged consultation (e.g. more than 1.5 years). Numerous key participants, including LegCo members, DC members and social service representatives, opined that consultation processes for establishing ICCMWs are time-consuming, making it difficult to gain support from residents and establish a consensus (Fig. 1), thereby delaying the establishment of ICCMWs. Although most government officials disagreed with establishing a formal, rigid time frame for public consultation, they agreed that the time frame for consultation should be ‘the sooner the better’. Others, however, believed that ample time is required for stakeholders to agree with ICCMW plans, public consultation is crucial, and the time required for public consultation should therefore be sufficient to enable residents to feel involved and understood.

Secondly, successful consultation processes often involved early support from DC members who explicitly expressed their support for the location selection plan to the general residents, lobby residents and residents connected with SWD and service providers. Key informants, including service providers, service users and government officials, agreed that DC members play a critical role in the success or failure of ICCMW consultation and location selection plans. They inform residents of the potential benefits of an ICCMW in the neighbourhood and act as a bridge between residents and the SWD or service providers in addressing residents’ concerns.

Thirdly, open and transparent public engagement activities make consultation processes more effective. In Hong Kong, the public has high expectations for the content and coverage of consultations on significant issues deemed related to vital public interests. Open consultation activities, such as community education campaigns, can effectively increase awareness of mental health issues and ICCMW services, collect public opinions and demonstrate the transparency of government policies and measures. However, some residents may misunderstand the purpose of public consultation. They may vote in opposition to ICCMW location selection plans. Therefore, clearly communicating the purpose and scope of engagement activities is essential.

Key informants had different perspectives on specific formats or mechanisms of public engagement and consultation, expressing a preference for face-to-face approaches. Members residing in the community tended to prefer resident forums in public consultations. However, government officials and elected members were more cautious about the potential fallout of this approach (e.g. intensification of conflicts and confrontation and minority rights not being well represented). However, approaches such as distributing consultation documents or information sheets by mail have not gained comprehensive support. According to one DC member, ‘there are so many advertisements nowadays. When residents open their mailboxes, they will dispose the leaflets right away. Also, the residents who are really interested actually prefer face-to-face replies rather than replying to us through questionnaires.’

Finally, several key informants, including resident representatives, volunteers and DC members, emphasised the importance of the government’s determination to establish ICCMWs despite public opposition successfully. After selecting a site, the government should facilitate the location selection process and avoid prolonged delays or withdrawal from the decision. The successful location selection cases indicated that government departments should proactively follow up with the opposition and address residents’ concerns instead of withholding or prolonging location selection plans.

### Approaches for reducing public opposition

Key participants identified various approaches that may be effective for reducing opposition to ICCMW establishment. These approaches focused on consultation length, location sites, communication between decision-makers and residents, promoting awareness of mental health, support and engagement by local decision-makers, collaboration between government departments and service providers and understanding neighbourhood characteristics. Firstly, regarding the length of consultations, numerous community members and government officials suggested that ICCMW plan proposals should be avoided during sensitive times, such as election periods. The reason is that location selection plans could be used as a political tool by election candidates.

Secondly, key participants suggested that open, wide-reaching, frequent and early communication on ICCMW location selection plans amongst DC members, residential representatives and residents is essential to smooth consultation processes. In addition to the details of location selection plans, the potential benefits and impacts of an ICCMW on a neighbourhood should be clearly explained to more effectively convince residents based on sufficient facts and logical arguments. Information on risk management and safety measures should be communicated to residents, including information on ICCMW users. All residents—not only key resident representatives—should be informed of location selection plans to ensure they feel respected and help secure trust. Specifically, information should be disseminated through neighbourhood newsletters, questionnaires and public notices outlining the mechanisms by which residents can express their concerns. Government departments and service providers should frequently communicate. Wide-reaching public engagement activities should begin as early as possible during the location selection process.

Thirdly, communication with residents through large-scale publicity should focus on improving their understanding of mental illness, increasing public support for ICCMW establishment. Therefore, public consultation should be conducted at the site selection stage and include the steps necessary to enhance residents’ understanding of mental illness. Fourthly, key participants reported that close collaboration amongst service providers, government departments, District Councillors and community leaders is critical for facilitating consultation processes and mitigating and resolving conflicts. In addition, many indicated that certain aspects of cooperation must be strengthened, including transparency, communication between parties and support from the government. Establishing ICCMWs requires SWD officials to perform strategic tasks, such as consulting with community members or influential community organisations (e.g. churches), inviting them to attend residential meetings and involving them in the community. Moreover, gaps in coordination may exist amongst government departments in terms of facilitating the selection of ICCMW locations. Specifically, one LegCo member cited a case where the SWD approved the proposal, but the Housing Authority later declined the lease application. This case illustrates that cooperation between government officials and between government officials and service providers must be strengthened.

Furthermore, key participants from ICCMW operating agencies, politicians and community members stressed that the responsibilities of different stakeholders should be clearly defined. Cooperation amongst government agencies, communication amongst government officials and other stakeholders in the community and support for service providers must be improved. Community lobbying and public engagement processes should not rely solely on service providers because NGOs cannot always clarify the details of services and policies on behalf of the government. Therefore, other government departments should assist the SWD and service providers to improve the general understanding of different features and issues related to districts and neighbourhoods.

Finally, government departments and service providers should understand the characteristics of proposed ICCMW sites. Careful investigation of neighbourhood profiles and dynamics amongst different stakeholders, cohesive engagement with influential figures and open and extensive consultation activities can effectively facilitate smooth public consultations for selecting the location of permanent ICCMW sites in a neighbourhood.

## Discussion

This study examined approaches for reducing public opposition to establishing community-based mental health facilities in Hong Kong (Table 2). In some cases, the research participants complained not being sufficiently informed about the nature of the potential users of and the scope of the mental health services provided. These situations echo the findings and suggestions of other studies for addressing public consultation strategies for mitigating the NIMBY effects [3, 4, 7, 37].

**Table 2:** Common factors for successful selection of ICCMW locations

1. Proactive support from local politicians throughout the entire process
2. Support from residents and their representatives
3. Strong determination of government officials to plan despite public opposition
4. Open and transparent public consultations and engagement activities

Different government departments and agencies should carefully study the characteristics of the neighbourhoods of planned ICCMWs. Improving the understanding of neighbourhood characteristics includes identifying key stakeholders, political and social dynamics and controversies and community demographics. To avoid discrepancies amongst different government agencies and minimise obstacles during the consultation process, after a suitable site for a permanent ICCMW is identified, a formal ‘task force’ should be established to determine effective consultation and engagement strategies and ensure that location selection and establishment process is conducted on time. This task force will be composed of the district offices of relevant government departments, the ICCMW service provider, District Council members and residents’ representatives.

**Fig. 2:**
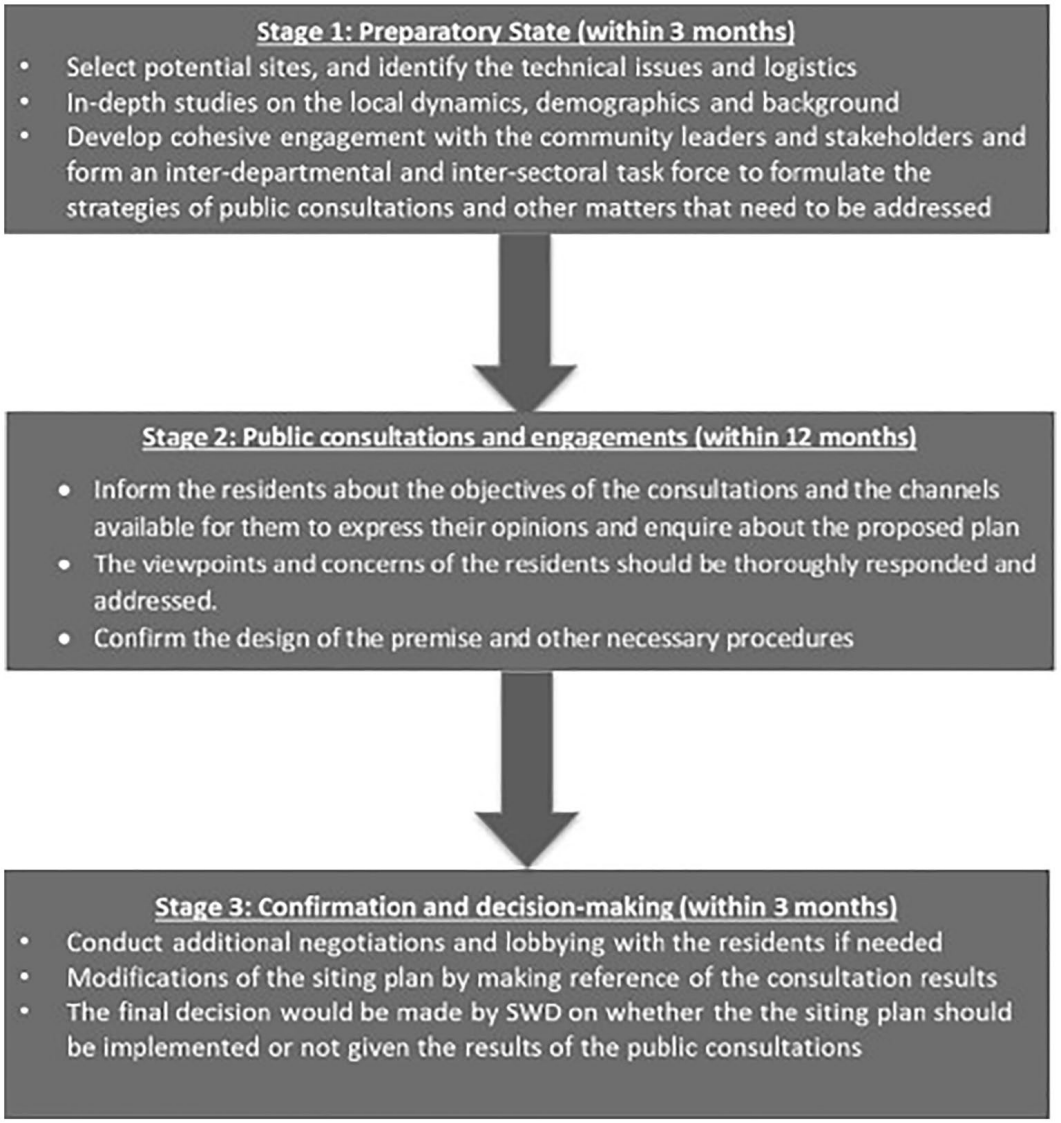
Three-stage public consolation protocol. This figure depicts the three-stage public consultation protocol for location selection of an ICCMW. The public consultation protocol should be developed to facilitate smooth and effective public consultations and should specify the time frame for each consultation and the target dates for confirming the ICCMW to avoid prolonged lobbying and delays in the establishment of services. Stage 1 (≤ 3 months) is the ‘preparatory stage’, which is initiated when a potential site has been identified. A task force led by district officers from the SWD or other government authorities and consisting of representatives from relevant departments should be established. Stage 2 involves public consultations and engagement activities, which should be completed within 12 months. When consulting the stakeholders, face-to-face approaches such as general residential meetings and other channels of communication are recommended to ensure that residents feel respected, receive more information, and are able to express their concerns. Stage 3 is the decision-making process. If the degree of local opposition is considerable, additional time and effort should be allocated for negotiations and community education. After the SWD has addressed various concerns by modifying the plan as needed, a decision should be made within 3 months

A public consultation protocol for the location selection of ICCMWs should be developed to facilitate smooth and effective public consultations, as shown in Fig. 2. The protocol should specify the time frame for each consultation process and the target dates for location selection to avoid prolonged lobbying and delays in establishing services. This protocol should stipulate that public consultations last no longer than 18 months and consist of three stages. Stage 1 would be the preparatory stage, which begins when a suitable site has been identified. At this stage, authorities should investigate local dynamics and inform community stakeholders of the plan. Stage 2 would consist of a series of public consultation and engagement activities involving face-to-face consultations approaches, including general residential meetings and other communication channels, to ensure that residents feel respected and can express their concerns. The final stage would be the actual decision-making process. After the authorities have addressed various concerns by modifying a plan as needed, a decision should be made within 3 months.

Hong Kong’s existing mechanisms for establishing sensitive facilities, such as ICCMWs, are similar to the practice of Macao and Taiwan by using negotiation with local residents. Community consultation and negotiations have been widely carried out in Hong Kong for decades and are able to enable members of the public to feel respected. Therefore, instead of eliminating all negotiation practices from the existing mechanism, public consultations should be retained in response to Hong Kong’s unique social context. The long-term roles and influence of community stakeholders and relationships between leaders and residents (as in the other Chinese societies) should be considered. However, some elements of human rights legislations and legal-based interventions should be adopted to facilitate the facility establishment process and to protect the interests of the residents.

Community education on mental health and receptiveness towards service users are important. Both subtle and explicit approaches must be implemented to help community residents understand and appreciate the users’ needs and former patients of mental health facilities. Previous studies on mental health services in Hong Kong indicated that people with mental illnesses were socialised to focus on the clinical definition of recovery. Local service users usually related recovery to restoring cognitive and social functioning prior to the onset of mental illness, which corresponds to functional recovery. The awareness and understanding of personal recovery were limited [28, 31, 38], leading to the persisting stigmatisation against mental health patients and ex-patients. Promotional and public engagement efforts in designated neighbourhoods should start as early as possible, ideally before public consultations. They should be implemented in different settings (e.g. schools and neighbourhoods), and these programmes should add emphasis on anti-discrimination and the rights of service users. These recommendations are consistent with those of Arboleda-Flórez and Stuart [1]. They suggested that advocacy approaches should be implemented to promote policy recognition, improve the quality of services and reduce social inequity.

## Conclusion

Given the difficulties involved with establishing ICCMW locations in Hong Kong, we cannot expect public consultations to obtain the endorsement of all community residents or stakeholders. Such efforts could severely inhibit the progress of location establishment plans. Any public consultation protocol established should specify the time frame for each consultation and the target dates for confirming ICCMW sites to avoid prolonged lobbying and delays in service establishment. Any ‘no-objection motions’ passed by resident organisations should not be a prerequisite for establishing an ICCMW is essential. Tenancy should be offered on the basis of the needs of the mental health service users but should not be delayed or blocked because of irrational stigma, stereotypes and discriminatory perspectives from the local residents and stakeholders.

## Data Availability

Data sharing is not applicable to this article because no datasets were generated or analysed for the current study.

## Abbreviations

NIMBY: ‘Not in my backyard’
ICCMW: Integrated Community Centre for Mental Wellness
SWD: Social Welfare Department
HKPSG: Hong Kong Planning Standards and Guidelines
DC: District Council
LegCo: Legislative Council
NGO: Nongovernmental organisation
